# Supplementary material for: High-Dose Methylprednisolone Pulses for 3 Days vs. Low-Dose Dexamethasone for 10 Days in Severe, Non-Critical COVID-19: A Retrospective Propensity Score Matched Analysis
Source: J Clin Med. 2021 Sep 28;10(19):4465. doi: 10.3390/jcm10194465 (PMC8509662; doi:10.3390/jcm10194465)
Supplement: Supplementary file 1 [file jcm-10-04465-s001.zip › jcm-1349753-supplementary.pdf]

Supplementary files

Table S1. Doses of methylprednisolone (MP) used

Dose of MP	n (%)
MP 100mg for 3 days	3 patients (1.6%)
MP 125mg for 3 days	170 patients (90%)
MP 250-500mg for 3 days	16 patients (8.4%)

Table S2. Percentage of missing data before multiple imputation

Lymphocytes	<5%
CRP	<5%
D-dimer	<5%
LDH	8%
Ferritin	20%
PaO2/FiO2	20%

Table S3. Risk factors of the combined outcome in the matched sample

	Univariate analysis		Multivariate analysis	
	OR (95%CI)	p-value	OR (95%CI)	p-value
Age/year	0.99 (0.98-1.01)	0.811	NS	
Gender (female)	0.85 (0.54-1.34)	0.491	NS	
BMI	1.01 (0.97-1.05)	0.726		
Smoking behavior				
Never smoker	1 ref.			
Former smoker	1.09 (0.69-1.72)	0.721		
Current smoker	1.63 (0.59-4.47)	0.343		
Degree of dependency				
None or mild	1 ref.			
Moderate	0.59 (0.25-1.39)	0.229		
Severe	2.69 (0.55-13.17)	0.220		
Arterial hypertension	1.04 (0.70-1.56)	0.838		
Dyslipidemia	1.26 (0.84-1.88)	0.266		
Diabetes mellitus	1.16 (0.71-1.90)	0.555		
Ischemic cardiopathy	0.63 (0.27-1.50)	0.300		
Cerebrovascular disease	0.62 (0.16-2.33)	0.477		
Dementia	0.97 (0.38-2.52)	0.956		
Chronic heart failure	0.91 (0.30-2.75)	0.863		
Chronic liver disease	0.89 (0.32-2.50)	0.820		
Severe chronic renal failure	0.64 (0.19-2.14)	0.470		
Cancer	1.18 (0.41-3.37)	0.762		
COPD	1.84 (0.74-4.59)	0.188		
Asthma	1.57 (0.28-8.68)	0.605		
OSAS	1.82 (0.77-4.30)	0.170		
PaO2/FiO2	0.98 (0.98-0.99)	<0.001	0.99 (0.98-0.99)	<0.001
Respiratory rate >20 bpm	2.70 (1.78-4.11)	<0.001	1.88 (1.17-3.02)	0.010
Lymphocytes/ x106/l	1.00 (1.00-1.00)	0.435		
CRP/ mg/l	1.01 (1.01-1.01)	<0.001	NS	
LDH/ U/l	1.01 (1.01-1.01)	<0.001	1.01 (1.01-1.01)	<0.001
Ferritin/ mcg/l	1.00 (1.00-1.00)	0.176		
D-dimer/ ng/ml	1.00 (1.00-1.00)	0.050	NS	
Steroids				
Bolus	1.65 (1.10-2.47)	0.016	NS	
Remdesivir	0.28 (0.07-1.08)	0.065	0.18 (0.04-0.80)	0.025
Tocilizumab	1.70 (1.13-2.57)	0.011	1.76 (1.09-2.84)	0.021

BMI: body mass index. NS: Not significant. COPD: chronic obstructive pulmonary disease. OSAS: obstructive sleep apnea syndrome. SOC: Standard of care CRP: C-reactive protein. LDH: lactate dehydrogenase.
